# How Does Clean Energy Consumption Affect Women’s Health: New Insights from China

**DOI:** 10.3390/ijerph19137943

**Published:** 2022-06-28

**Authors:** Fanghua Li, Abbas Ali Chandio, Yinying Duan, Dungang Zang

**Affiliations:** 1 College of Economics, Sichuan Agricultural University, Chengdu 611130, China; lfhua@stu.sicau.edu.cn (F.L.); alichandio@sicau.edu.cn (A.A.C.); 2 School of Business & Tourism, Sichuan Agricultural University, Chengdu 611180, China; duanyinying@sicau.edu.cn

**Keywords:** cleaner household energy, women’s health, CHARLS, IV-O-Probit model, energy and health poverty, mediating and moderating effects

## Abstract

The United Nations (UN) has identified the promotion of cleaner energy and improving women’s health as two important elements in achieving the global sustainable development goals. However, the impact of household clean energy consumption on women’s health needs to be further analyzed and improved based on new methods, new data, and new perspectives. This paper used the data from the 2018 China Health and Retirement Longitudinal Study as the sample, and the Ordered Probit model, the instrumental variable (IV) approach, the conditional mixed process (CMP) method, and the mechanism analysis model were applied to empirically investigate the impact of cleaner household energy consumption on women’s health. The findings are the following: (1) It is found that cleaner household energy consumption improved women’s health, and after selecting “respondent’s regions of residence” as an IV to overcome endogenous issues, the estimated results remained significant. (2) The mechanistic estimation showed that air quality, social contact, and well-being play a mediating role in the effects of cleaner household energy consumption on women’s health, while digital ability plays a moderating role in the cleaner household energy consumption impact on women’s health. (3) This study further explored that cleaner household energy consumption significantly reduced the likelihood of women being diagnosed with hypertension, hyperlipidemia, cancer, lung disease, asthma, and depression. The conclusion of this paper that “cleaner household energy can enhance the level of women’s health” supports the viewpoints of some present literature. At the same time, this paper puts forward four policy recommendations based on the research conclusions.

## 1. Introduction

Women’s health can be harmed by long-term household use of non-clean energy sources such as firewood, coal, and cow dung [1]. Households in developing countries (regions) such as Southeast Asia [2], South Africa [3], the Sahara [4], and China [5,6] have used unclean energy for a long time, which has caused more obvious harm to women’s health. The use of non-clean energy sources increases women’s risk of respiratory [2], cardiovascular, and psychological [3] diseases and also increases maternal and infant mortality [4,5]. As a result, improved women’s health and cleaner household energy have become important aspects of the Sustainable Development Goals (SDGs) [6,7].

Clean energy in the household reduces the cost of time spent by women on tasks such as cooking and heating, and women get more rest and have fewer physical illnesses [8,9]. It also frees up time for women to participate in social activities; women can relieve mental stress by socializing [10] and gain respect and opportunities to improve mental health [11]. Non-clean energy burning pollutes indoor air [12], and women’s prolonged exposure to polluted air triggers a health crisis [13]. Cleaner household energy eliminates harmful emissions such as CO_2_, NO_2_, PM (Particulate Matters), etc., [14] and optimizes indoor air quality and reduces the likelihood of women being diagnosed with respiratory diseases [15,16]. The indoor living environment and the outdoor ecosystem have both improved with the increasing use of clean energy in the household [17]. Improved indoor and outdoor environmental quality can reduce disease generation and transmission, thereby enhancing women’s health [18]. At the same time, the use of clean energy reduces gender inequality [19], educational inequality [20], and household vulnerability [21] and significantly increases women’s life satisfaction and well-being [22], thus improving women’s disease prevention and health awareness [23]. Stable electricity supply and long-term household use of electricity can promote cleaner household energy, thereby reducing women’s health vulnerability [24].

A part of the study focused on the choice of household energy consumption and its impact on women’s health in developing countries (regions). Akter and Pratap [25] collected energy data from over 9,000 households in India, and analysis showed that household use of LPG significantly improves women’s health benefits. More than 75% of households in Pakistan still use solid non-clean energy, and by comparing the health of households using clean and non-clean energy, a significant improvement in women’s health was found in households using clean energy [26]. Data from sub-Saharan Africa indicates that the use of modern clean energy in households provides health security for women and children [27]. Wang et al. [28] found that from 2000 to 2014, the amount of clean energy consumed in China continued to increase while maternal mortality declined, demonstrating a significant negative association between cleaner household energy and maternal mortality. Using data from the China Family Panel Studies (CFPS) as a sample, Wu [29] selected self-assessment of health as the explanatory variable to investigate the impact of household cooking fuel choice on women’s health along several dimensions and found that cleaner household energy was more significant in improving the health of women over 46 years of age.

**Figure 1:**
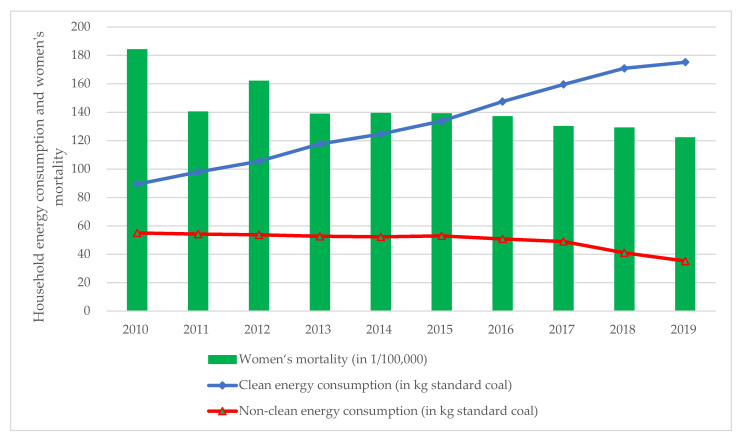
Women mortality due to three prevalent diseases and per capita household energy usage in China (2010–2019). **Data source:** China National Statistical Yearbook (2011–2020); **Note:** The 3 prevalent diseases include respiratory illnesses, psychological illnesses and pregnancy illnesses.

The current literature argues that the long-term use of non-clean energy in the home can be detrimental to women’s health and that cleaner energy in the home can help improve women’s health. Furthermore, macro statistics for China support the views of the present literature. This paper collects data on per capita household energy consumption in China from 2010–2019, including clean energy consumption (electricity + LPG + natural gas) and non-clean energy consumption (coal + coal gas). From 2010 to 2019, data on deaths due to respiratory diseases, psychological diseases, and pregnancy diseases were also collected for Chinese women because existing studies indicate that household energy consumption is associated with these three categories of diseases [3,28,30]. As can be seen from the line graph in Figure 1, Chinese household clean energy consumption is increasing year on year, while non-clean energy consumption continues to decrease. Clean energy consumption is now greater than non-clean energy consumption, which indicates a shift towards cleaner household energy consumption in China. This conclusion was demonstrated by Ellison et al. [31] who collected data on the energy consumption of 753 households in China. The statistics show a gradual shift from solid non-clean fuels to clean fuels in Chinese households. The histogram in Figure 1 shows an overall decreasing trend in women’s mortality from all ages due to three diseases. Therefore, based on Figure 1, this paper concludes that household energy clean-up can improve women’s health.

However, macro statistics are limited in that they do not count the number of Chinese households using firewood, grass, cow dung, or biogas as their main source of energy. In fact, there are many households in rural China that use fuelwood, cow dung, or biogas. The 2018 China Health and Retirement Longitudinal Study (CHARLS) is the largest and most comprehensive micro-study on households and health in China. Therefore, this paper uses data from the 2018 CHARLS as a sample and explores the impact of cleaner household energy on women’s health. We reviewed some literature related to this paper, and we found that the limitations of the current literature are: (1) China is one of the countries with the highest energy consumption in the world, but there is still a lack of research on household energy consumption and women’s health based on the micro-data from China; (2) Many studies do not deal with potential endogeneity issues in the section of empirical analysis; (3) Many studies only analyze whether energy consumption affects women’s health without further research on how energy consumption impacts women’s health through mechanisms analysis; (4) Many studies only analyze the relationship between energy consumption and women’s overall health and do not explore the impact of energy consumption on common diseases in women in more detail. Therefore, the innovations of this paper include: (1) using a large sample of microdata from China to analyze the impact of energy consumption on women’s health; (2) using the instrumental variables approach and the conditional mixed process estimation method to deal with potential endogenous issues; (3) taking different microdata to test the robustness of the empirical results; (4) using a mediating effects model and a moderating utility model to analyze the mechanism of the impact of cleaner household energy on women’s health; (5) discussing the impact of cleaner household energy on hypertension, asthma, depression, and eight other common diseases. The remaining sections include data and methods (Section 2), empirical analysis (Section 3), mechanistic analysis (Section 4), further research (Section 5), and discussion, conclusions, and policy recommendations (Section 6).

## 2. Data and Method

### 2.1. Data

**Figure 2:**
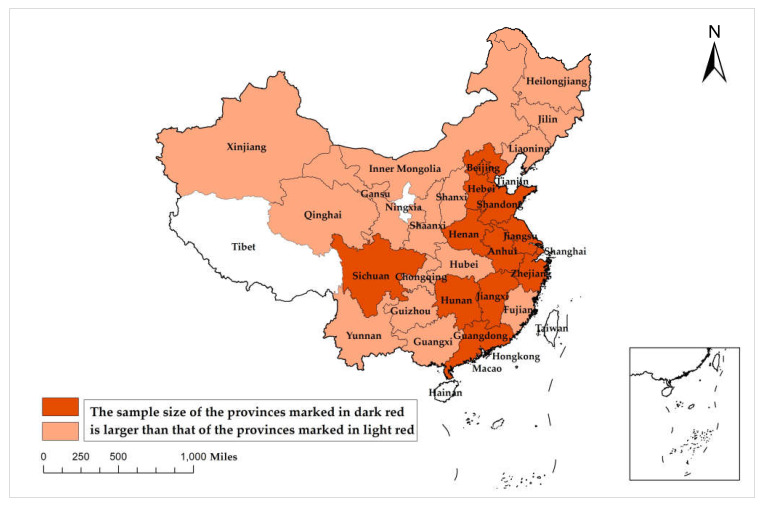
Sample distribution of the 2018 CHARLS. **Source:** CHARLS National Baseline Survey 2013 User Manual. **Note:** China now has 34 provinces (municipalities/autonomous regions/special administrative regions), and CHARLS 2018 covers 28 provinces, excluding Hong Kong, Macau, Taiwan, Hainan, Tibet, and Ningxia. Since CHARLS does not announce the specific sample size of each province, the sample size of different provinces can only be distinguished by the shade of color.

This study used data from the 2018 CHARLS as a sample of the empirical and mechanism analysis sections. CHARLS is the earliest and most comprehensive survey project reflecting data on family health and aging in China [32]. The CHARLS covered 28 provinces (Figure 2), 150 cities (districts/counties), and 450 villages/urban communities in China. A total of 10,257 household samples and 17,708 individual sample data were obtained. The survey covers basic information about the respondents, health status and function, cognition and depression, health care and insurance, and household economic status. The data were first matched cross-sectionally across the modules; then extreme and missing values were processed, and the data were normalized to give a total dataset of 5,125 for analysis.

### 2.2. Variables

#### 2.2.1. Response Variable: Women’s Health (WH)

The commonly used measure is the subjective health evaluation as a proxy variable for women’s health [29,33], which is also referenced in this paper. Therefore, the data of the survey question *“What do you think about your health?”* was chosen to measure women’s health.

#### 2.2.2. Explanatory Variable: Cleaner Household Energy (CHE)

According to the questionnaire *“What is the main fuel used for cooking in your home?”* The answers were: coal, straw, firewood, natural gas, biogas, LPG, and electricity. If the respondent chose clean energy, we set CHE = 1; if the respondent chose non-clean energy, we set CHE = 0.

#### 2.2.3. Control Variables

This paper focuses on the effect of CHE on WH, while some individual and household factors may have a causal relationship with WH. Therefore, more individual and household factors that may influence WH need to be included in the empirical model r to effectively analyze the relationship between CHE and WH. With reference to Liu et al. [34], this paper selected several control variables in terms of individual respondent information, household economic status, and household infrastructure such as age, income, and house structure. Information on the main variables in this paper is reported in Table 1.

### 2.3. Variable Descriptive Statistics

In this paper, the mean, standard deviation, and maximum and minimum values of the main variables were calculated using Stata v15.0 software The results from Table 2 show the description of individual characteristics of the women in the study sample. While there has been a gradual improvement in the health of Chinese women, there is still a substantial percentage (25%) of women with poor health. The majority of the women reported fair health conditions (44%). More than two-thirds of the households now use CHE (69.23%), illustrating the shift toward cleaner household energy consumption. The majority of the women were middle-aged (mean = 49.18 years), with 70.83% of respondents aged between 41 to 60 years. The surveyed women had a low level of education, as evident by the fact that 97.52 percent had only completed high school. Most of the women were married (77.19%) and had high public health insurance coverage (96.16%). There exists a high disparity in terms of income and expenditure, but most of the respondents were debt free. Around 86% of the households surveyed had concrete, steel, brick, and wood as a BS, indicating improved living conditions for women. Most of the respondents had flushing toilets (63.96%), an indicator of an improved sanitary environment for women. The respondents were mostly living in rural settings (69.91%) and were more satisfied (84.27%) with the air quality, indicating an improvement in household air quality. However, a significant proportion of women (47.61%) are still not involved in social contact activities, and a large majority (54.54%) of women did not have the digital ability but show a better sense of well-being (89.04%) and think they are happier, indicating that women’s life satisfaction has improved.

### 2.4. Models

The response variable WH in this paper is an ordered multi-categorical variable, and for this type of data, a commonly used econometric model is the Ordered Probit model [35,36,37]. Hence, this paper constructs Ordered Probit (O-Probit) models (Equations (1) and (2)) on CHE and WH and used *Stata v 15.0* software for empirical analysis.

(1) $$WH_{i}^{*}=\omega_{n}+\beta_{n}\times CHE+\varphi_{n}\times CV_{r}+\mu_{k}$$

(2) $$WH^{*}=\left\{\begin{array}{c} 1\;if\;0<i\leq 1\\ 2\;if\;1<i\leq 2\\ 3\;if\;2<i\leq 3\\ 4\;if\;3<i\leq 4\\ 5\;if\;4<i\leq 5 \end{array}\right.$$

The $WH_{i}^{*}$ is the latent variable of health; WH=1, 2, 3, 4, 5 denotes five self-evaluations of health; $\omega_{n}$ is the intercept term; $\beta_{n}$ and $\varphi_{n}$ are regression coefficients; CHE is cleaner household energy; $CV_{r}$ is the control variable; and $\mu_{k}$ denotes the error term.

The dynamic linkages between cleaner household energy and women’s health may be influenced by other factors, i.e., there may be mediating and moderating mechanisms between *CHE* and *WH*. Therefore, to examine the mediating mechanism between cleaner household energy and women’s health and referring Wen et al. [38], this paper sets up the mediating effect model as (Equation (3)):

(3) $$\left\{\begin{array}{c} WH_{i}^{*}=\omega_{n}+\beta_{n}\times CHE+\varphi_{n}\times CV_{r}+\mu_{k}\;\\ MV=\omega_{1}+\beta_{1}\times CHE+\varphi_{1}\times CV_{r}+\mu_{1}\;\\ WH_{i}^{*}=\omega_{2}+\beta_{2}\times CHE+\rho \times MV+\varphi_{2}\times CV_{r}+\mu_{2} \end{array}\right.$$

The MV is the mediating variable, and ρ is the regression coefficient of the mediating variable. If $\beta_{n}$, $\beta_{1}$, $\beta_{2},$ and ρ are all significant, it means that MV has a mediating effect on CHE and $WH_{i}^{*}$.

Moreover, with reference to Wen et al. [38], set up the moderating effect model (Equation (4)):

(4) $$\left\{\begin{array}{c} WH_{i}^{*}=\omega_{n}+\beta_{n}\times CHE+\varphi_{n}\times CV_{r}+\mu_{k}\;\\ WH_{i}^{*}=\omega_{3}+\gamma \times RV+\varphi_{3}\times CV_{r}+\mu_{3}\;\\ WH_{i}^{*}=\omega_{4}+\theta \times \left(CHE\times RV\right)+\varphi_{4}\times CV_{r}+\mu_{4}\; \end{array}\right.$$

where RV is the moderating variable, and γ and θ are the regression coefficients. If $\beta_{n}$, γ, and θ are all significant, then RV has a moderating effect on the CHE impact on $WH_{i}^{*}$.

In the present regression model, we have taken into account the multi-collinearity problem that could arise between different explanatory variables and control variables. We used the variance inflation factor (VIF) to measure the multi-collinearity issue. The result shows a VIF of 5.21, which is less than 10, indicating that the model did not suffer multi-collinearity issue [39].

## 3. Empirical Analysis and Discussion

### 3.1. Basic Regression

Table 3 model (1) reports the regression results for cleaner household energy and women’s health, and model (2) shows the average marginal effects at five cut-off points. The results from model (1) show that cleaner household energy is positively (significantly) associated with women’s health, indicating that cleaner household energy improves women’s health. The trend in the average marginal effect in model (2) also shows that women’s health improves with the shift to cleaner household energy.

The relationship between the control variables and women’s health is also reported in Table 3. ***Age*** is significantly and negatively associated with women’s health, indicating that as women get older, their physical functions and resistance to disease decline, and their health deteriorates [40]. ***Marriage*** significantly (positive) impacts women’s health, illustrating that a good marital relationship can improve women’s physical and mental health [41]. Likewise, better ***education*** significantly improves women’s health as women with some years of formal education are more likely to be better informed about the harmful impacts of the use of non-clean fuel and other nutritional needs of their own [42]. Furthermore, ***income*** also significantly enhances women’s health, demonstrating that higher income is associated with a higher willingness to pay for a healthier lifestyle, and a greater ability to prevent and control diseases [18]. On the other hand, ***debt*** (significantly) negatively impacts women’s health because debt repayment reduces investment in health and could also lead to psychological stress [43]. In addition, the use of a ***flushable toilet*** in the home can have a positive impact on women’s health by reducing the creation and spread of disease and improving the hygienic environment and air quality in the home [44].

In this paper, the empirical analysis results of expenditure, MI, BS, and WH did not pass the significance test, indicating that MI and BS have no significant effect on women’s health. However, we observe that the regression coefficient of experience and WH is −0.005 (<0), which means that the increase in household economic expenditure may be detrimental to women’s health; the regression coefficients of MI, BS, and WH are 0.033 (>0) and 0.004 (>0), respectively, illustrating that participation in medical insurance and safer housing structures may be beneficial for improving women’s health. Generally, we only analyze and discuss significant results in depth in the paper.

### 3.2. Robustness Check

In this paper, robustness tests are conducted using replacement sample data. The China Family Panel Studies *(**CFPS**)* is a national, comprehensive social tracking survey reflecting the social, economic, demographic, and educational changes in China. Household energy consumption and health are the main focus of CFPS. The Chinese General Social Survey *(**CGSS**)* is the first nationwide, comprehensive and continuous academic survey project in China that systematically and comprehensively collects data at the social, community, household, and individual levels. Household energy use and health are also an important part of CGSS. However, the present large-scale micro-survey data in China are missing samples from Tibet, where energy consumption differs from other areas [45]. The China Tibetan Livelihood Development Survey *(**CTLDR**),* which began in 2018, focuses on investigating the changing lives of Tibetan residents in China, with a more complete collection of data on the health and energy use of Tibetan farmers and herders. The CTLDR is able to overcome the shortcomings of CHARLS, CFPS, and CGSS. This paper follows the same methodology for measuring the main variables and uses data from the 2018 CFPS, CGSS, and CTLDR to again validate the nexus between cleaner household energy and women’s health. The results are reported in Table 4.

Based on the outcomes for the CFPS (model 2), CGSS (model 3), and CTLDR (model 4), it is verified that cleaner household energy significantly positively affected women’s health, and the trend in the mean marginal effect results demonstrates that household use of clean energy can gradually improve women’s health (see Table 4). The findings of this study are aligned with previous studies such as Zhang et al. [9] and Li et al. [46].

### 3.3. Endogeneity: Instrumental Variables Approach and CMP Estimation (IV-O-Probit Model)

Omission of variables and sample selection bias can lead to endogenous issues, which can cause errors in the empirical results; however, endogenous issues in empirical analysis cannot be completely avoided. Therefore, “Region of residence of respondents (Regions, 1 = rural, 2 = urban-rural combination, 3 = urban)” was chosen as the instrumental variable and the IV-O-Probit model was used to address possible endogenous issues. The reason for choosing “Regions” as the instrumental variable is that households in different regions use different amounts of energy due to differences in resource endowments [27,47], which demonstrates that the region of residence is directly related to whether the household uses clean energy but does not have a direct effect on women’s health [48]. This theoretically satisfies the basic requirements of the instrumental variable. The instrumental variables meet the requirements at the theoretical level.

There are currently technical barriers to adding instrumental variables to the O-Probit model for direct estimation. A common approach when dealing with endogenous issues in O-Probit models is to use a conditional mixed process estimation containing instrumental variables [49]. The results of the IV-O-Probit model and CMP estimation for the endogenous issues are reported in Table 5.

The results of models (1) and (2) in Table 5 show that the instrumental variable “Regions” is significantly and positively associated with the explanatory variable CHE but not with the explanatory variable WH. The auxiliary estimated parameter *atanhrho_12* is significantly different from 0 (*p*-value = 0), indicating that there is a significant correlation between the two equations in the joint cubic equation model and that adopting the CMP estimation method is more effective than estimating them separately, also indicating that CHE is an endogenous variable. Models (3) and (4) show the results of the CMP estimates and mean marginal effects of the IV-O-Probit model, illustrating that after overcoming endogenous issues, cleaner household energy still significantly improves women’s health with a coefficient of 0.0932 greater than 0.0611 in the basic regression, implying that the basic regression underestimates the extent of the impact of clean energy use on women’s health. The average marginal effect at the five cut-off points is also greater than the value in the basic regression, again validating the result that cleaner household energy can gradually enhance the level of women’s health. The *F-statistic* for the first stage is 185.3, which is greater than the experimental value of 10, demonstrating that there is no problem of weak instrumental variable.

## 4. Mechanism Analysis

The results of the empirical analysis suggest that cleaner household energy can significantly improve women’s health, but no specific mechanisms of impact are indicated. The mechanism analysis based on the empirical analysis will further investigate how cleaner household energy affects women’s health. Mechanism analysis in economics generally consists of a mediating effects analysis and a moderating effects analysis. The purpose of studying mediating effects is to explore the internal factors that influence the relationship between cleaner household energy and women’s health, while moderating effects analysis is to explore the external factors that influence this relationship [50].

### 4.1. Testing for Mediating Effects of Air Quality (AQ), Social Contact (SC) and Well-Being (WB)

This paper used women’s satisfaction with indoor air quality as a proxy variable for air quality (AQ) because women have a direct ability to perceive indoor air quality; therefore, women’s satisfaction with indoor air quality can reflect the real situation of AQ to some extent [51]. At the same time, in this paper, we examined whether AQ, SC, and WB played a mediating role in the effect of cleaner household energy (CHE) on women’s health (WH). The result was reported in (Table 6).

The results of models (1), (2), and (3) in (Table 6) illustrate that CHE significantly (positive) influences AQ and that better AQ significantly improves WH, which means that AQ plays a mediating effect on CHE impact on WH [2]. CHE will reduce harmful gas emissions [13], optimize indoor AQ, and reduce the exhalation of harmful gases into women’s bodies, thus improving health. The results of models (1), (4), and (5) in (Table 6) suggest the existence of a mediating mechanism whereby CHE increases SC frequency and enhances WH [11]. Clean energy has the advantage of being efficient, and its use saves time for women to engage in SC, which can reduce mental stress and therefore reduce the incidence of mental illness [11]. The results of the mediating effect tests for WB are reported in models (1), (6), and (7) in (Table 6). It is verified that CHE significantly increased women’s WB, and it was significantly and positively allied with WH. Finally, the corresponding *Soble* test and *Bootstrap* test results indicate that AQ, SC, and WB have partially mediating effects between CHE and WH.

### 4.2. Testing for Digital Ability Moderation Effects

We used the data from the question “Do you usually use WeChat? (1 = yes, 0 = no)” to measure digit ability (DA) and then examine whether DA plays a moderating role in the effect of CHE on WH. The results are shown in Table 7. The findings of model (2) in Table 7 show that digital ability significantly contributes towards women’s health. Furthermore, the result of model (3) reveals the interaction term between CHE, DA, and WH, illustrating that DA has a moderating role in the relationship between CHE on WH and reinforces the positive impact of CHE on WH.

Digital ability (DA) refers to the ability of residents to access information using digital devices and to use technology and information for their own benefits. Digital technologies can significantly change the way households produce and operate, increase household income [52], and accelerate the clean-up of household energy. Women can also use the Internet to learn about energy diversification [53] and increase their willingness to use clean energy. Women can also use the Internet to learn energy use techniques and methods to improve energy efficiency. Thus, digital ability can increase the chances of cleaner household energy.

## 5. Further Research

In recent years, chronic diseases such as hypertension, hyperlipidemia, diabetes, cancer, lung disease, stroke, asthma, and psychological disorders such as depression have become a global health concern with increasing diagnosis and mortality rates [54,55]. Deaths from chronic diseases accounted for 88.5% of all deaths in China in 2019, with 80.7% of deaths from cardiovascular diseases, cancer, and chronic respiratory diseases [56]. Existing studies have found that households using non-clean energy are at risk of cardiovascular disease, respiratory disease, and psychological disorders [16,30].

In the present study, we refer to the study of Zhang et al. [57], select data from seven research questions and use a factor analysis model to measure the depression index as a proxy variable for depression. The seven statements include: *(1) I had trouble keeping my mind on what I was doing; (2) I felt depressed; (3) I felt everything I did was an effort; (4) I felt hopeful about the future; (5) I felt fearful; (6) I was happy; and (7) I felt lonely”*. The response to each statement is *“1 = rarely or none of the time, 2 = some or a little of the time, 3 = occasionally or a moderate amount of the time, 4 = most or all of the time”*.

The current study further explores the nexus between cleaner household energy and chronic disease and depression in women, and the results are reported in Table 8. The results of models (1), (2), (4), (5), and (7) show that cleaner household energy significantly reduces the probability of women being diagnosed with hypertension, hyperlipidemia, cancer, lung disease, and asthma. In addition, the findings of model (8) indicate that cleaner household energy is negatively associated with depression at a significant level of 0.01, implying that a shift towards cleaner household energy could improve women’s mental health.

## 6. Discussion, Conclusions and Policy Recommendations

### 6.1. Discussion

Cleaner household energy (CHE) and women’s health (WH) are two hot topics of current global concern. Some of the current literature discusses the positive impact of CHE on WH, and these findings provide a valuable reference for this paper. Based on the analysis of micro-data from China, this paper finds that CHE significantly improved WH, which supports the views and conclusions of Belmin et al. [24], Akter and Pratap [25], and Li et al. [46], etc. The contributions of this paper include: (1) We provide the first analysis of the impact of CHE on WH using four micro-datasets from China, the results of which validate the reliability of some of the existing literature conclusions and provide a new reference for future research. (2) This paper analyses not only the direct effects of CHE on WH but also the mediating and moderating effects of air quality, social contacts, well-being, and digital ability in the effects of CHE on WH, the mechanisms of which have been analyzed in only a few papers. (3) The paper also analyses the impact of CHE on common chronic diseases and depression in women, adding to the novelty of the findings. However, there are limitations to this paper: (1) Due to many psychological and social uncertainties, there may be errors in the health data provided by women during the research process, which may affect the quality of the data. This problem can be solved by optimizing the research methods and increasing the number of respondents. (2) This analysis was conducted using micro-data from China, and the conclusions obtained may be more applicable to China or developing countries (regions). Therefore, in the new study, data from both developing and developed countries should be collected for comprehensive and comparative analysis to draw conclusions of global value.

### 6.2. Conclusions

This study examined the dynamic nexus between cleaner household energy consumption and women’s health in the context of China. The main findings of this paper revealed that cleaner household energy significantly improved women’s health. We further verified the estimated outcomes by using the data of China Family Panel Studies (CFPS, 2018), the Chinese General Social Survey (CGSS), and the China Tibetan Livelihood Development Survey (CTLDR) and proved that cleaner energy plays a vital role and impressively enhances the better health status of women in the country. In addition, the estimated results showed that air quality, social contacts, and well-being partially mediate the effects of cleaner household energy on women’s health, while digital ability has a moderating effect on cleaner household energy impacts on women’s health. Finally, it is also proven that utilization of clean household energy can significantly reduce the likelihood of women being diagnosed with hypertension, hyperlipidemia, cancer, lung disease, asthma, and depression.

### 6.3. Policy Recommendations

Based on the findings, this study provides the following policy suggestions:

- *First,* the government should accelerate the exploitation and conversion of clean energy resources (i.e., wind, solar, hydro, natural gas, etc.) to build a diversified clean energy supply system in the country.
- *Second,* the government should improve energy transportation facilities and household energy-burning facilities to enhance the supply capacity of clean energy and increase the willingness to use it.
- *Third,* the government should be empowering women, increasing women’s decision-making power in family and social matters, and reducing gender inequalities in health care.
- *Fourth,* the government should improve the medical infrastructure by increasing the number of hospitals, upgrading the level of medical care and services, and improving the capacity for disease prevention and treatment.

## Figures and Tables

**Table 1:** Variable’s selection and definition.

Variables’ Type	Name	Definition
Response variable	Women’s Health (WH)	What do you think about your health? 1 = very poor; 2 = poor; 3 = fair; 4 = good; 5 = very good.
Explanatory variable	Cleaner household Energy (CHE)	What is the main source of cooking fuel in your household? Natural-gas, marsh gas, liquefied petroleum gas and electric = clean energy = CHE = 1; coal, crop residue, and wood burning = non-clean energy = CHE = 0.
Control variables	Age	2018—Year of birth.
	Education	What is the highest level of education you have now (not including adult education)? 1 = illiterate; 2 = did not finish primary school, home school or elementary school; 3 = middle school, high school, vocational school, or associate degree; 4 = bachelor’s degree, master’s degree, or doctoral degree.
	Marriage	What is your marital status? 0 = never married; 1 = married; 2 = widowed, divorced and separated (don’t live together as a couple anymore).
	Medical Insurance (MI)	Have you bought medical insurance? (Include public medial insurance and private commercial medical insurance), 0 = no; 1 = yes.
	Income	*Ln* (annual income) = *Ln* (wage income + business income + transfer income + property income + 1). unit: RMB
	Expenditure	*Ln* (annual expenditure) = *Ln* [(monthly expenditure) × 12 + 1]. unit: RMB
	Debt	*Ln* (bank loan debt + credit card debt + other debt). unit: RMB
	Building Structure (BS)	What type of structure is this building? 1 = stone; 2 = Mongolian yurt/woolen felt/tent; 3 = cave dwelling; 4 = wood/thatched; 5 = adobe; 6 = concrete and steel/bricks and wood.
	Flushable Toilet (FT)	Does your household use a flushable toilet? 0 = no, 1 = yes.
Instrumental variable	Regions	Region of residence of respondents? 1 = rural, 2 = urban-rural combination, 3 = urban.
Mediating variables	Air Quality (AQ)	Women’s satisfaction with indoor air quality, 1 = not at all satisfied, 2 = not very satisfied, 3 = somewhat satisfied, 4 = very satisfied, 5 = completely satisfied.
	Social contact (SC)	Have you participated in social activities in the recent month? Yes = 1, No = 0.
	Well-being (WB)	Self-life satisfaction, 1 = not at all satisfied, 2 = not very satisfied, 3 = somewhat satisfied, 4 = very satisfied, 5 = completely satisfied.
Moderating variable	Digital ability (DA)	Do you usually use WeChat? 1 = yes, 0 = no.

**Table 2:** Variable’s description statistics.

Variable	Observations	Proportion	Mean	Std. Dev.	Min	Max
WH	5125	100.00%	3.10	1.03	1	5
WH = 1	302	5.89%				
WH = 2	990	19.32%				
WH = 3	2277	44.43%				
WH = 4	996	19.43%				
WH = 5	560	10.93%				
CHE	5125	100.00%	0.71	0.45	0	1
CHE = 1	3548	69.23%				
CHE = 0	1577	30.77%				
Age	5125	100.00%	49.18	10.64	18.00	97.00
Age = 18~40	120	2.34%				
Age = 41~50	2624	51.20%				
Age = 51~60	1006	19.63%				
Age = 61~70	853	16.64%				
Age = 71~97	522	10.19%				
Education	5125	100.00%	2.16	0.79	1	4
Education = 1	1101	21.48%				
Education = 2	2209	43.10%				
Education = 3	1688	32.94%				
Education = 4	127	2.48%				
Marriage	5125	100.00%	1.21	0.43	0	2
Marriage = 0	41	0.80%				
Marriage = 1	3956	77.19%				
Marriage = 2	1128	22.01%				
MI	5125	100.00%	0.96	0.19	0	1
MI = 1	4928	96.16%				
MI = 0	197	3.84%				
Income	5125	100.00%	8.99	2.63	0.00	15.43
Expenditure	5125	100.00%	8.91	1.81	0.00	13.34
Debt	5125	100.00%	1.56	3.75	0.00	14.93
BS	5125	100.00%	5.76	0.85	1	6
BS = 1	121	2.36%				
BS = 2	60	1.17%				
BS = 3	39	0.76%				
BS = 4	49	0.96%				
BS = 5	409	7.98%				
BS = 6	4447	86.77%				
FT	5125	100.00%	0.64	0.48	0	1
FT = 1	3278	63.96%				
FT = 0	1847	36.04%				
Regions	5125	100.00%	1.52	0.83	1	3
Regions = 1	3583	69.91%				
Regions = 2	432	8.43%				
Regions = 3	1110	21.66%				
AQ	5125	100.00%	3.22	0.84	1	5
AQ = 1	162	3.16%				
AQ = 2	644	12.57%				
AQ = 3	2440	47.61%				
AQ = 4	1653	32.25%				
AQ = 5	226	4.41%				
SC	5125	100.00%	0.52	0.50	0	1
SC = 1	2685	52.39%				
SC = 0	2440	47.61%				
WB	5125	100.00%	3.31	0.81	1	5
WB = 1	157	3.06%				
WB = 2	404	7.88%				
WB = 3	2520	49.17%				
WB = 4	1787	34.87%				
WB = 5	257	5.01%				
DA	5125	100.00%	0.45	0.50	0	1
DA = 1	2330	45.46%				
DA = 0	2795	54.54%				

Data source: The raw data was processed using Stata v15.0 software: WH = women’s health; CHE = cleaner household energy.

**Table 3:** The regression results of CHE and WH.

	O-Probit (1)	O-Probit (2) Average Marginal Effect				
Variables	WH	WH = 1	WH = 2	WH = 3	WH = 4	WH = 5
CHE	0.061 **	−0.007 **	−0.012 **	−0.007 **	0.010 **	0.012 **
	(0.029)	(0.003)	(0.006)	(0.003)	(0.004)	(0.005)
Age	−0.010 ***					
	(0.001)					
Education	0.069 ***					
	(0.010)					
Marriage	0.098 *					
	(0.059)					
MI	0.033					
	(0.035)					
Income	0.017 ***					
	(0.003)					
Expenditure	−0.005					
	(0.004)					
Debt	−0.006 *					
	(0.003)					
BS	0.004					
	(0.009)					
FT	0.062 ***					
	(0.017)					
Observations	5125	5125	5125	5125	5125	5125

Note: Robust standard errors in parentheses *** *p* < 0.01, ** *p* < 0.05, * *p* < 0.1. WH = woman’s health, 1 = very poor, 2 = poor, 3 = fair, 4 = good, 5 = very good; CHE = cleaner household energy, 1 = clean energy, 0 = non-clean energy; MI = medical insurance; BS = building structure; FT = flushable toilet.

**Table 4:** Robustness test results for replacement sample data.

	CHARLS_2018	CFPS_2018	CGSS_2018	CTLDR_2018
	O-Probit (1)	O-Probit (2)	O-Probit (3)	O-Probit (4)
Variables	WH	WH	WH	WH
CHE	0.061 **	0.075 ***	0.160 ***	0.085 **
	(0.029)	(0.025)	(0.026)	(0.041)
	**Average marginal effect**			
WH = 1	−0.007 **	−0.020 ***	−0.014 ***	−0.013 **
	(0.003)	(0.007)	(0.002)	(0.007)
WH = 2	−0.012 **	−0.006 ***	−0.033 ***	−0.026 **
	(0.006)	(0.002)	(0.005)	(0.011)
WH = 3	−0.007 **	0.002 ***	−0.016 ***	−0.010 **
	(0.003)	(0.001)	(0.003)	(0.005)
WH = 4	0.010 **	0.009 ***	0.023 ***	0.034 **
	(0.004)	(0.003)	(0.004)	(0.016)
WH = 5	0.011 **	0.014 ***	0.041 ***	0.037 **
	(0.005)	(0.005)	(0.007)	(0.018)
CV	Control	Control	Control	Control
Observations	5125	7346	6353	295

Note: Robust standard errors in parentheses *** *p* < 0.01, ** *p* < 0.05. WH = woman’s health, 1 = very poor, 2 = poor, 3 = fair, 4 = good, 5 = very good; CHE = cleaner household energy, 1 = clean energy, 0 = non-clean energy; CFPS_2018 = the 2018 China Family Panel Studies data; CGSS_2018 = the 2018 Chinese General Social Survey data; CTLDR_2018 = the 2018 China Tibetan Livelihood Development Research data; CV = control variables.

**Table 5:** The results of IV-O-Probit model for endogenous issues.

	First Stage		CMP Estimation Method					
	O-Probit (1)	Probit (2)	IV-O-Probit (3)	IV-O-Probit (4) (Marginal Effect)				
Variables	WH	CHE	WH	WH = 1	WH = 2	WH = 3	WH = 4	WH = 5
CHE	0.061 **		0.093 **	−0.005 **	−0.012 **	−0.004 **	0.011 **	0.019 **
	(0.029)		(0.037)	(0.002)	(0.005)	(0.002)	(0.005)	(0.009)
Regions	0.023	0.141 ***						
	(0.019)	(0.045)						
atanhrho_12(P)			0.000	0.000	0.000	0.000	0.000	0.000
F-statistics	185.3							
CV	Control	Control	Control	Control	Control	Control	Control	Control
Observations	5125	5125	5125	5125	5125	5125	5125	5125

Note: Standard errors in parentheses *** *p* < 0.01, ** *p* < 0.05. WH = woman’s health, 1 = very poor, 2 = poor, 3 = fair, 4 = good, 5 = very good; CHE = cleaner household energy, 1 = clean energy, 0 = non-clean energy; REGIONS = regions where the respondent lives? 1 = rural, 2 = urban-rural combination, 3 = urban; CV = control variables.

**Table 6:** The results of mediating effect test for CHE and WH: AQ, SC, and WB.

	O-Probit (1)	O-Probit (2)	O-Probit (3)	Probit (4)	O-Probit (5)	O-Probit (6)	O-Probit (7)
Variables	WH	AQ	WH	SC	WH	WB	WH
CHE	0.061 **	0.142 ***	0.073 **	0.319 ***	0.070 ***	0.139 ***	0.072 **
	(0.029)	(0.034)	(0.036)	(0.039)	(0.026)	(0.034)	(0.034)
AQ			0.044 ***				
			(0.016)				
SC					0.064 **		
					(0.032)		
Happiness							0.041 **
							(0.017)
Soble test (*p*)		0.021 < 0.05		0.062 < 0.10		0.033 < 0.05	
Bootstrap (500)		Direct effect (*p* = 0.017 < 0.05)		Direct effect (*p* = 0.033 < 0.05)		Direct effect *p* = 0.041 < 0.05	
		Indirect effect (*p* = 0.038 < 0.05)		Indirect effect (*p* = 0.048 < 0.05)		Indirect effect *p* = 0.031 < 0.05	
CV	Control	Control	Control	Control	Control	Control	Control
Observations	5125	5125	5125	5125	5125	5125	5125

Note: Robust standard errors in parentheses *** *p* < 0.01, ** *p* < 0.05. WH = woman’s health, 1 = very poor, 2 = poor, 3 = fair, 4 = good, 5 = very good; CHE = cleaner household energy, 1 = clean energy, 0 = non-clean energy; AQ = air quality, 1 = not at all satisfied, 2 = not very satisfied, 3 = somewhat satisfied, 4 = very satisfied, 5 = completely satisfied; SC = social contact = social contact in recent month, 1 = yes, 0 = no; WB = well-being = self-life satisfaction, 1 = not at all satisfied, 2 = not very satisfied, 3 = somewhat satisfied, 4 = very satisfied, 5 = completely satisfied; CV = control variables.

**Table 7:** The results of the moderation effect test for CHE and WH: DA.

	O-Probit (1)	O-Probit (2)	O-Probit (3)
Variables	WH	WH	WH
CHE	0.061 **	0.063 **	0.075 **
	(0.029)	(0.030)	(0.037)
DA		0.067 **	0.062 **
		(0.028)	(0.029)
CHE * DA			0.041 **
			(0.020)
CV	Control	Control	Control
Observations	5125	5125	5125

Note: Robust standard errors in parentheses ** *p* < 0.05. WH = woman’s health, 1 = very poor, 2 = poor, 3 = fair, 4 = good, 5 = very good; CHE = cleaner household energy, 1 = clean energy, 0 = non-clean energy; DA = digital ability = Do you usually use WeChat? 1 = yes, 0 = no, respondents choose “1” who has digital ability, vice versa; CV = control variables.

**Table 8:** The regression results of CHE and different diseases.

	Probit (1)	Probit (2)	Probit (3)	Probit (4)	Probit (5)	Probit (6)	Probit (7)	OLS (8)
Variables	Hypertension	Hyperlipidemia	Diabetes	Cancer	Lung	Stroke	Asthma	Depression
CHE	−0.108 ***	−0.148 ***	0.016	−0.006 **	−0.177 ***	0.019	−0.218 ***	−0.111 ***
	(0.015)	(0.015)	(0.059)	(0.003)	(0.015)	(0.015)	(0.015)	(0.021)
CV	Control	Control	Control	Control	Control	Control	Control	Control
Observations	5125	5125	5125	5125	5125	5125	5125	5125

Note: robust standard errors in parentheses *** *p* < 0.01, ** *p* < 0.05. CHE = cleaner household energy, 1 = clean energy, 0 = non-clean energy; Hypertension = are you diagnosed with hypertension? 1 = yes, 0 = no; Hyperlipidemia = are you diagnosed with hyperlipidemia? 1 = yes, 0 = no; Diabetes = are you diagnosed with diabetes? 1 = yes, 0 = no; Cancer = are you diagnosed with cancer? 1 = yes, 0 = no; Lung = are you diagnosed with lung disease? 1 = yes, 0 = no; Stroke = are you diagnosed with stroke? 1 = yes, 0 = no; Asthma = are you diagnosed with asthma? 1 = yes, 0 = no; Depression = depression index calculates by the factor analysis model; CV = control variables.

## Data Availability

The datasets used or analyzed during the current research are available from the corresponding author on reasonable request.
